# Human Infants Detect Other People's Interactions Based on Complex Patterns of Kinematic Information

**DOI:** 10.1371/journal.pone.0112432

**Published:** 2014-11-19

**Authors:** Martyna A. Galazka, Laëtitia Roché, Pär Nyström, Terje Falck-Ytter

**Affiliations:** 1 Uppsala Child and Babylab, Department of Psychology, Uppsala University, Uppsala, Sweden; 2 Institut National de la Santé et de la Recherche Médicale (INSERM), Unit 930, Tours, France; 3 University François-Rabelais of Tours, Unit 930 ‘Imaging et Brain’ - Team 1 Autism, Tours, France; 4 Center of Neurodevelopmental Disorders at Karolinska Institutet (KIND), Stockholm, Sweden; University of Montreal, Canada

## Abstract

Do infants perceive other people's interactions by means of a mechanism that integrates biological motion information across the observed individuals? In support of this view, the present study demonstrates that infants (*N* = 28, Age  = 14 months) discriminate between point light displays representing disrupted and non-disrupted interactions between people, even though the two interaction types are identical at the level of individual point light agents. Moreover, a second experiment (sample 2: *N* = 28, Age  = 14 months) indicated that visual preference in this context is influenced by an audiovisual integration processes that takes into account the presence of an interaction between people. All these results were found exclusively for upright displays – when stimuli were shown upside-down (disrupting biological motion processing), performance was random. Collectively, these findings point to an important role for biological motion in social perception in human infants.

## Introduction

The ability to identify biological motion – the distinct, non-rigid movement patterns produced by humans or animals – is believed to serve important evolutionary purposes, including detection of predators and filial attachment. A substantial body of research shows that adults are highly efficient in processing biological motion information [1]–[5].

Whether and how humans use biological motion to understand the interactions of other people is far less clear than how biological motion contributes to understanding the actions of single individuals. This topic has, however, received increased attention in recent years [6]–[9]. In an influential study, Neri, Luu, and Levi (2006) [10] presented adults with point-light displays of two agents that were either dancing or fighting. In these interactions, the actions of one agent clearly constrain the actions of the other agent. For example, when one agent extended his hand to punch another, the second agent moved backward to avoid the hit. In one (synchronized) condition, the interaction remained unaltered. In another (desynchronized) condition, the temporal relationship between the two agents was manipulated, disrupting their interaction. In addition, the authors created single agent versions of these stimuli, by scrambling one of the agents. When judging whether one or two agents were present, participants were most accurate when meaningful, synchronized interactions were shown, despite the fact that even the desynchronized displays contained two agents. The authors interpreted this finding as reflecting implicit knowledge about human interactions and the effects one agent's actions have on the other agent's actions. A similar study manipulated the relative position of the two agents rather than the timing of the actions, and found that for upright point-light presentations, the interaction strength was rated higher for non-disrupted interactions than for pairs in which the agents' position was switched [11]. Manera et al. (2011) extended Neri et al.'s findings, suggesting that even without any physical contact between agents, the gestures of one agent enhanced visual perception of the other agent's biological motion.

Sensitivity to biological motion information from single individuals is early emerging in development. Preferential looking paradigms have shown that when displays of biological motion are presented along side inverted displays or random point-light motion (both of which disrupt biological motion processing) [12]–[13], infants as young as 2 days spontaneously orient to the original, non-disrupted biological motion displays [14]–[15]. At 6 months infants are able to distinguish directionality of a point-light walker [16] and perceive it as a solid form [17]. That is, infants renewed their interest in the video stimuli when the point-light walker changed walking direction or seemingly walked through a solid structure. Within the first year, infants also show different neural responses to upright versus inverted [18] or scrambled point-light displays [19]. Hirai & Hiraki (2005) suggested that the greater ERP amplitude in response to human biological motion compared to scrambled motion reflects activity of the occipitotemporal region, likely including the superior temporal sulcus (STS). Biological motion perception has been related to the posterior STS in adults as well [20]–[22]. In addition, this region responds to a wide range of other social stimuli, and has an important role in multimodal (e.g, audiovisual) information processing [23]–[25].

So far, only one developmental study has included stimuli with more than one point-light agent. Centelles et al. (2013) presented adults and children between 4 and 10 years with displays of two point-light individuals that were either interacting (e.g. conventional social situations, emotional situations, or games) or were engaged in intransitive actions with no interaction component (e.g. rotating the trunk, jumping, raising a leg). The children were then asked to report for each stimulus if an interaction was present. It was found that at 4 years children performed above chance and by 7 years performance was comparable to that of the adults. Of importance, however, is that in this study, the non-interacting and interacting pairs were not derived from the same movement recordings. Consequently, it is not possible to exclude that the children based their conclusion about the presence of interaction on the information contained in an individual agent without relating information across the two agents in the pair. In other words, seeing someone point may make a person infer the presence of an interaction between people without actually processing information from more than one person. Thus, currently it is not known whether children or infants are able to detect human interaction when this information can be derived only by integrating biological motion information from multiple agents (processing one agent's movements in relation to the other agent's movements), or if they are restricted to processing each individual separately.

**Figure 1:**
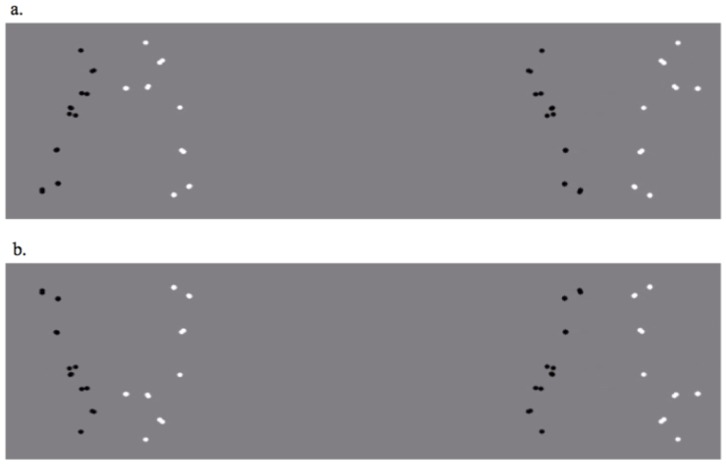
Stimuli (from Falling Action) examples illustrating the experimental manipulations (a) the Upright Condition with the non-disrupted pair on the left, disrupted pair on the right and (b) the Inverted Condition, where the same stimuli were shown upside-down. In the non-disrupted pair, the black-point-light agent fell backwards *toward* the white point-light agent. The white-point agent then caught the black-point-light agent before she hit the ground and pushed her back to standing position. In the disrupted pair, the black-point-light agent fell *away from* the white point-light agent in the disrupted pair, and the rescue act by the white agent was not in concordance with the position of the black agent. This sequence was repeated twice during the 9 second trial.

To address this question, a preferential looking paradigm was used to examine 14-months-old infants' preferential attention to a non-disrupted pair of two interacting agents presented side-by-side a pair of the same agents but with a disrupted interaction. The disruption of an interaction was achieved by manipulating the two agents' orientation relative to each other (see Figure 1 and methods; see also [11]). These stimuli were piloted on a group of adult university students, which confirmed a preference for the interacting pair (*M* = 0.595, *SD*  = .125; *t*(13) = 2.837, *p* = .014, *d* = 1.57 one sample t-test against 0.5). Because inversion disrupts biological motion processing [26]; [12]–[13] we included control stimuli in which the same displays (each containing one non-disrupted and one disrupted interaction) were shown upside down. We used non-invasive eye tracking technology to measure viewing performance.

In Study 1, we predicted that 1) children would prefer to look at the side of the screen containing non-disrupted interaction rather than the disrupted interaction, and 2) that this preference should be evident only when the displays were shown in an upright orientation.

## Study 1

### Methods

#### Participants

The sample consisted of twenty-eight 14-month-old infants (*M* = 430 days; *SD*  = 11.87 days; 13 female). Two additional infants were tested but were excluded from the final sample due to an insufficient gaze recording by the eye tracker (less than 20% of gaze samples).

All participants were recruited from live birth records and only the parents who had indicated an interest in participating in research with their child were contacted to participate. Infants were primarily Caucasian and from a middle-class background. Parents received a gift voucher worth approximately 10 euros for their participation. The study was conducted in accordance with the 1964 Declaration of Helsinki and all the parents provided written consent according to the guidelines specified by the Uppsala Ethical Review Board in Uppsala, Sweden (Etikprövning av forskning som avser människor), which has granted the permission to conduct this research.

#### Apparatus

Eye movements were recorded using a Tobii T120 (Tobii Technology Inc., Stockholm, Sweden) remote near-infrared eye-tracker (gaze was recorded at 60 Hz). An integrated 33.7 cm×27 cm monitor was used to present the stimuli. This system has a reported accuracy of 0.5 visual degrees and freedom of head movement within 30×22×30 cm. A standard 5-point infant calibration was used and passed by all participants [27].

#### Stimuli

We recorded two different types of interactive actions between two actors using motion-tracking technology (Qualisys Motion Capture Systems, Göteborg, Sweden). For each actor, 15 reflective markers were used and were positioned on each actor's head (1), shoulders (2), elbows (2), wrists (2), hips (2), knees (2), ankles (2) and feet (2). A 6-camera motion capture set up was used to identify and track the motion of the markers.

The actors performed two types of interactive actions. In the first (Falling action), one actor was standing behind the other. After approximately 2 seconds, the actor in front fell backwards without bending the knees or hips. The second actor, standing behind, caught the first actor and pushed her back to a standing position. This action sequence was repeated twice in each trial (naturally repeating, no clipping). In the second type of interactive action (Pushing action), the actors were standing side-by-side (separated by a distance of approximately 1 meter) facing the same direction. Each actor had one arm extended, toward the other actor. With the extended arm, the actors were attempting to make the other actor loose balance by pushing and dragging the other's hand. Of note, although in reality the actors were holding hands, due to the position of the marker on the hands, the point-light agents were spatially separated in this recording.

The stimuli videos were created from text files exported from the motion capture software, using an in-house MATLAB program (MathWorks Inc., Natick, MA). In order to distinguish the two agents from each other, one agent's point-lights were changed to black, while the second agent's point-lights were changed to white both against a grey background (see Figure 1). The angle of the (virtual) camera was set optimally for each action. For the Falling action, the camera saw the agents in profile view. In the Pushing action the agents were facing the camera, to clearly show their attempts to pull and push each other with their arms.

Each video stimulus contained two pairs of agents (i.e. four individuals). On one side of the screen, the original, non-disrupted interaction was shown. On the other side, each point-light agent was flipped horizontally, so that the agents in the disrupted pair were facing in the opposite direction than their ‘peer’ in the non-disrupted pair. This manipulation preserved each agent's individual movements, but effectively disrupted the interaction between them. Critically, the two point-light pairs presented in each stimulus were created from the same motion capture recording, and thus had identical low level features within individual agents. However, when the agents in the non-disrupted pair were moving away from each other, the agents in the disrupted pair were moving toward each other and vice versa. We positioned the two agents in the disrupted pair in such a way, that their mean distance matched the mean distance between the agents in the non-disrupted display (across all frames in the stimulus video). The inter-agent distance was operationalized as the distance between the hips of the two agents. Visual inspection by several raters suggested that this matching worked well also for the global impression of the distance between the agents (see Video S1 and Video S2, online).

The study included two conditions. In the Upright Condition, these displays were presented upside-up and in the Inverted Condition, the same set of video stimuli was presented upside-down.

**Figure 2:**
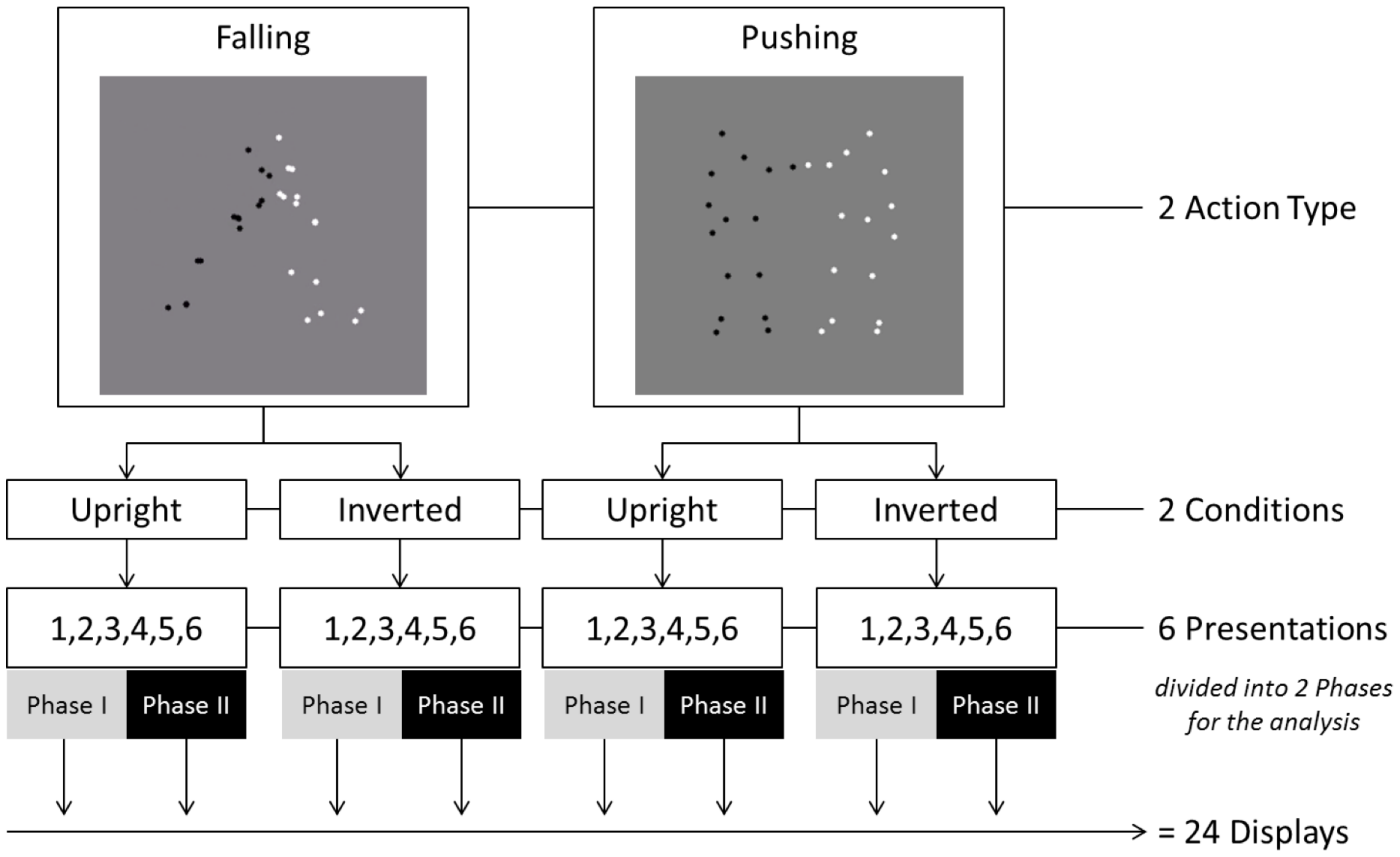
The chart describes stimuli number and distribution according to Action Types, Conditions and Phases.

In total, each child observed 24 trials (12 in the Upright and 12 in Inverted Condition) (Figure 2). Each condition contained 6 trials from each Action Type (see above). Each stimulus lasted 9 seconds (total presentation time of 3 min 36 sec). The two conditions were presented in a pseudo-random order (with left-right counterbalancing).

#### Procedure

Following written consent from the caregivers, all infants and parents entered a dimly lit and quiet testing room with a chair placed in front of the eye-tracker. The parents were asked to sit in the chair and children were directed to sit on their parent's lap, approximately 60 cm from the monitor. By instruction and monitoring, the experimenter ensured that parents were sitting still and did not communicate with their child during the stimulus presentation. The importance of not moving during the stimulus presentation was stressed before starting the session. It was also controlled that the eyes of the parent was outside the tracking space of the eye tracker.

In order to maintain the participant's attention throughout the video presentations, various ‘attention grabbers’ preceded each experimental stimulus. The attention grabbers used were various colourful geometric patterns paired with an attractive sound. Each attention grabber lasted about 5 seconds.

#### Data reduction and analysis

For all the stimulus types, three rectangular areas of interest (AOIs) were created: screen AOI and two half-screen AOIs. The Screen AOI encompassed the entire screen (29.3×24.2 visual degrees) and the two half-screen AOIs (15.6×24.2 visual degrees) covered the two pair types (non-disrupted versus disrupted) to determine preference for a pair type. The two half-screen AOIs covered the entire screen and there was no distance between them.

Data were analysed from the entire trial sequence (9 sec). A trial was excluded from the analysis if looking time at the Screen AOI was less than 2 sec (22%). Looking time at each AOI was calculated using TimeStudio (www.timestudioproject.com), a MATLab© program specifically designed for analysing time series data. We used a fixation filter with the velocity threshold of 35 pixels window and the distance threshold of 35 pixels (Tobii Fixation filter).

#### Statistical analysis

The dependent variable was the proportion of looking at the non-disrupted pair divided by the looking time at both the non-disrupted and the disrupted pairs. This measure of preference for the non-disrupted interaction automatically controls for differences in total looking times between conditions. In Study 1, the independent variable was condition (Upright vs. Inverted), Alpha level  = .05 throughout.

### Results

**Figure 3:**
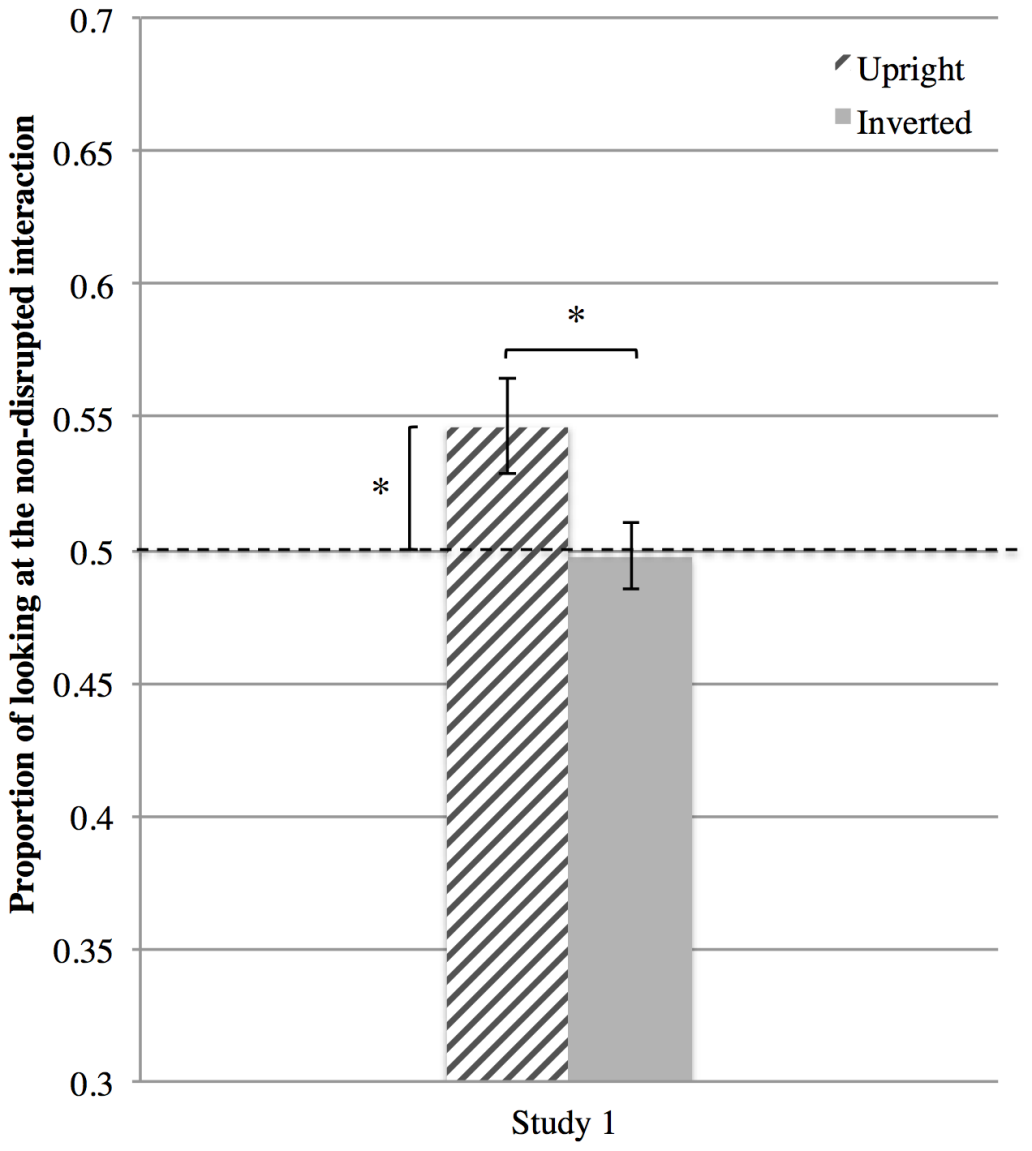
Proportion of looking at the non-disrupted interaction the Study 1 in the Upright and Inverted Conditions. Looking preference was calculated as looking duration to the non-disrupted display divided by looking duration at both non-disrupted and disrupted displays. *  = *p*<.*05*. Error bars show standard error of the mean.

The infants oriented preferentially to the non-disrupted pair in the Upright (*M* = .55, *SD*  = .09; *t*(27) = 2.56, *p* = .016, *d* = 0.985, one sample t-test, tested against 0.5), but not in the Inverted Condition (*M* = .49, *SD*  = .07, *t*(27) = −.181, *p* = .858, *d* = 0.069 one sample t-test, tested against 0.5). A paired sample t-test revealed a significant difference between the proportion of looking at the non-disrupted interaction in the Upright and Inverted Condition, *t*(27) = 2.14, *p* = .041, *d* = 0.824 (Figure 3). On average, infants looked at the screen for 6.11 sec (*SD*  = 1.29 sec) in the Upright Condition, and 5.93 sec (*SD*  = 1.36 sec) in the Inverted Condition.

### Discussion - Study 1

The results of Study 1 supported both our predictions; infants oriented preferentially to the non-disrupted interaction in the Upright Condition, and the difference between the Upright and the Inverted Conditions was significant. This suggests that 14-month-old infants are able to integrate biological motion information from multiple individuals.

Study 1 included several steps aimed at reducing the possibility of confounding variables. First, the inclusion of an Inverted Condition, and the fact that this manipulation blocked the preference for the interacting pair, suggest that the main finding is related to biological motion processing rather than lower level cues. Moreover, the properties (e.g. velocity profiles of the markers, relative position of the markers) of each individual agent were identical between the disrupted and non-disrupted pairs, which contrasts the current work with previous studies [6], [28]. Finally, we adjusted the distance between the two agents in the disrupted pair to correspond to the distance between the agents in the non-disrupted pair in order to rule out distance between agents as a confounder.

Study 2 was included to further strengthen the conclusion that the effect was not driven by lower level stimulus properties, but rather, the disruption of the interaction between the two agents in the pair.

### Study 2

The same visual stimuli were presented to a new group of infants, but in Study 2 the stimuli were accompanied by an audio recording of an interaction between two people (two voices talking to each other; see Methods below). From early on, infants are able to integrate what they see with what they hear – a process believed to be crucial for their perception of a unified, multimodal world [29]–[31]. Events that are redundantly specified across multiple senses tend to recruit attention and become perceptually salient. Research has shown that very young infants attend and discriminate more readily between novel stimuli when presented with synchronous sounds and sights than when presented with either a unimodal or desynchronized stimulus [32]–[34]. Although these studies used non-reduced visual stimuli, other work has shown that infants integrate auditory and visual information even when the visual stimulus is a point-light display [35]–[36]. Critically, in Study 2, the presence of an interaction between two people was the only common attribute shared by the soundtrack and the non-disrupted pair of the visual stimulus.

We predicted that hearing two people talking to each other would affect concurrent visual preference for the non-disrupted pair in the Upright condition. In the Inverted condition (where recognition of biological motion is impaired), we expected similar performance to Study 1 (random looking). This pattern would imply that visual preference in this context is influenced by audiovisual integration processes, taking into account the presence of an interaction between people.

### Methods

#### Participants

Participants were twenty-eight 14-month-olds (*M* = 438 days; *SD*  = 5.93; 12 female). An additional 5 infants were tested but were excluded from the final data set due to insufficient gaze recording by the eye tracker (*N* = 2) or due to fussiness that prevented the completion of the study (*N* = 3). Samples in Study 1 and Study 2 were not overlapping.

#### Stimuli

The stimuli videos were identical to those from Study 1, with the exception that we added an auditory recording of two people interacting. The content of the two voices was tailored to the visual content of each action. Specifically, as one of the actors began falling backwards a voice said ‘*I am falling*!’. As the second actor caught the falling actor, another voice said ‘*You're okay now*’ in response, as the falling actor was pushed back into upright position (see Video S3, online) This was repeated to correspond to the repetition of the action in the visual stimulus. For the second Action Type, where the actors were standing side-by-side attempting to make the other actor loose balance by pushing and dragging the other's hand, the voices said ‘*Don't push me*!’ while the other answered ‘*Yes, I want to*!’ (see Video S4, online). This conversation was repeated two times during stimulus presentation. Note that even if this conversation matched the visual content of the stimulus, it was the presence of two voices engaging in a conversation per se, rather than the specific semantic content, that was expected to be important, given the infants' young age.

Procedure, data reduction and analytic procedures were identical to Study 1 unless otherwise specified.

### Results

In the Upright Condition, the infants gave similar preferential attention to the non-disrupted and the disrupted pairs (preference for non-disrupted pairs was.49; *SD*  = .08). Similarly, in the Inverted Condition, the infants gave similar preferential attention to the two pairs (*M* = .49, *SD*  = .07). The proportion of looking at the non-disrupted interaction was not significantly different between the conditions (*t*(27) = −.001, *p* = .999, *d*<.001; paired samples t-test). Looking at the screen AOI was similar in the two conditions (*M* = 7.00 seconds, *SD*  = 1.33 in the Upright orientation and *M* = 7.02 seconds, *SD*  = 1.38 in the Inverted orientation).

These results suggest that adding the auditory stimulus eliminated the preference for the non-disrupted pair seen in Study 1. Before concluding this, however, it is important to evaluate some alternative explanations. In particular, previous research has documented that visual preference can change, typically from familiarity preference to novelty preference, across experimental trials [37]–[38]. Such dynamic shifts in preference can easily be confused with random looking or lack of discrimination. To check whether a preference change was present in Study 2, we split the looking data into two phases. Phase I encompassed Trials 1–3 for each Action Type (Falling; Pushing) in each Condition (Upright; Inverted), while Phase II encompassed Trials 4–6 for each Action Type in each Condition (Figure 2).

**Figure 4:**
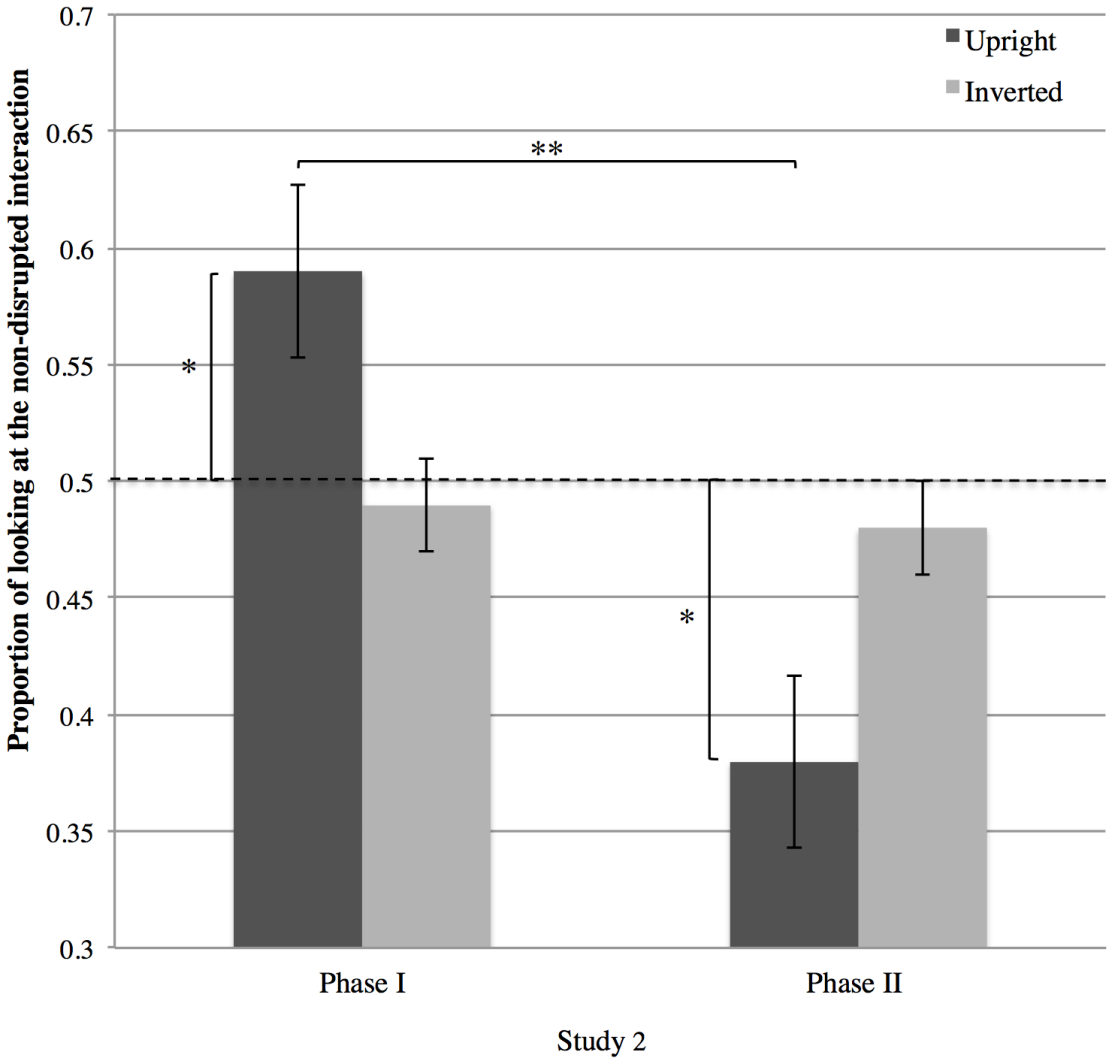
Proportion of looking at the non-disrupted interaction across in the Upright and Inverted Conditions (Study 2). Study 2 included auditory cues signaling the presence of an interaction between people. Initially (Phase I), infants attended preferentially to the non-disrupted pair, but with time (Phase II) preference switched to the disrupted pair. This change was only found in the Upright Condition. Looking preference was calculated as looking duration to the non-disrupted display divided by looking duration at both non-disrupted and disrupted displays.* = *p*<.05; ** = *p*<.*01*. Error bars show standard error of the mean.

A 2(Condition: Upright, Inverted) ×2(Phase: I, II) repeated measures ANOVA revealed a significant Condition by Phase Interaction (*F*(1, 26) = 5.88, *p* = .023, η^2^ = 0.184) (Figure 4). Post hoc tests revealed that in Phase I in the Upright Condition, infants oriented towards the non-disrupted pair (*M* = .58, *SD* = .19), (*t*(27) = 2.18, *p* = .038, *d* = 0.839), mimicking the result of Study 1. In Phase II, however, they oriented preferentially towards the disrupted display (*M* = .38, *SD*  = .21), (*t*(26) = −3.09, *p* = .005, *d* = 1.21), and the change from Phase I to Phase II was statistically significant, (*t*(26) = 2.98, *p* = .006, *d* = 1.17). In the Inverted Condition, no such change was observed (*t*(26) = 0.57, *n.s.*). Specifically, in the Inverted Condition, looking preference in Phase I was.49 (*SD*  = .11) which is not significantly different from chance (*t*(27) = 0.139, *n.s.*). The same was found for Phase II in the Inverted Condition (*M* = .48, *SD*  = .14; *t*(27) = 0.823, *n.s.*).

In light of this finding, we checked whether similar effect of Phase was present in Study 1. We found no effect of Phase In Study 1, neither in the Upright (*F*(1, 26) = 0.206, *n.s.*) or the Inverted Condition (*F*(1, 26) = 0.155, *n.s.*). To test formally whether Phase influenced looking differently in the two studies, we submitted the data from the Upright Condition to a 2(Study: 1, 2) by 2(Phase: I, II) ANOVA, confirming that an interaction effect between these two factors (*F*(1, 52) = 6.416, *p* = .014, *η^2^* = .110). Thus, adding the soundtrack modulated looking preference (across phases).

Supplementary analyses (see Analysis S1, online) found no effect of Action Type, and replicated the effect of Phase using more than two phases. Analysis S1 also provides analyses of absolute (rather than relative) looking measures for the different AOIs.

### Discussion - Study 2

In the Upright Condition in Study 2, infants looked preferentially towards the non-disrupted interaction in the early trials but this preference reversed as a function of time. As expected, the change in preference was only found in the Upright Condition – in the Inverted Condition no preference was found in any phase. This pattern speaks against the involvement of lower-level processes, as they should not be selectively linked to the Upright Condition.

Previous studies have indicated that whether infants prefer to look at a novel or a familiar stimulus depends on the amount of time they have had inspecting the familiar stimulus beforehand, as well as the stimulus complexity [39]. With brief familiarization time with a stimulus, infants prefer to look at this stimulus when subsequently paired with an unfamiliar stimulus. However, if infants are given more familiarization time, they tend to look at the unfamiliar stimulus rather than the familiar stimulus [38]. Thus, infants show a familiarity preference if they have not fully encoded the stimulus, but when they have enough exposure to fully encode it they give preferential attention to the unfamiliar stimuli [37]–[38], [40]–[43].

The current study is different from the above-cited studies in that it only included paired visual preference trials, with no prior familiarization trials. Nevertheless, the striking shift in preference introduced by the soundtrack suggests that the multimodally and redundantly specified interaction in Study 2 facilitated encoding of the non-disrupted stimulus, causing infants to switch their attention to the less familiar, disrupted pair towards the latter part of the experiment.

## General Discussion

Prior research has demonstrated infants' striking ability to integrate motion information from multiple markers representing the movements of one single person. The current work demonstrates that human infants integrate biological motion information from multiple individuals. This conclusion is supported by two key findings from Study 1: i) the infants showed evidence of visual preference for non-disrupted rather than disrupted pairs, even though these pairs were matched in terms of single-agent motion patterns, and ii) these effects were found for upright presentations only (inversion, known to disrupt biological motion processing, significantly modulated the effects).

Moreover, the modulation by speech in Study 2 suggests that audiovisual integration processes that takes into account the presence of two interacting individuals are influencing visual preference in this context. Evidence for preferential looking was exclusively found for upright presentations in Study 2 as well. Unlike in Study 1, in Study 2, we observed a change in preference from non-disrupted to disrupted pairs across experimental trials. As argued above, this indicates that speech signalling an interaction between two people speeds up the encoding of the (audiovisual) non-disrupted pair [37]. The results of Study 2 are in line with a large body of research demonstrating that compared to unimodal events, multimodally specified events recruit selective attention that in turn facilitate learning in young infants [32]–[35]. Indeed, because the visual stimuli displayed two interacting point light agents and the soundtrack included voices from two people interacting, the presence of an interaction was redundantly specified in both the visual and auditory modality in Study 2. Thus, taken together, the current work points to several levels of information integration in human infants: i) the integration of information of individual point-lights to a percept of a point-light person, ii) the integration of information from two point-light persons into a percept of their interaction, and iii) the integration between interaction cues presented in the visual and auditory modalities.

An important aspect of the current study was that the non-disrupted and disrupted pairs were created from the same recording of two actors' movements. Consequently, at the level of individual agent's movements, the two sides were identical (this entails that even single-agent audiovisual properties were matched across the sides). Any valid explanation of the result must relate to a property that resulted from the spatial manipulation of the two agent's relative positions we introduced experimentally. While we cannot completely rule out that biological motion (upright presentation) facilitated detection of a lower level visual property which differed between the two pairs in Study 1, and which in turn was perceptually amplified by the soundtrack in Study 2 [44], it is hard to imagine such an undefined residual property, in particular one that would produce similar effects across two qualitatively different Action Types (Falling and Pushing) (see Analysis S1). Indeed, for both Action Types, we observed similar effect of Phase, and we found no statistically different effect involving the factor Action Type in neither Study 1 nor Study 2. The current study shows that the infants do not only discriminate between the non-disrupted and the disrupted pairs, they also prefer to look at non-disrupted interaction (although with time, they may start to inspect the disrupted pair). Thus, it seems that the results describe a mechanism that allows infants to orient their attention to interactions between people, rather than to people acting individually, detached from others. Strikingly, this mechanism seems to require only biological motion information to be activated.

This attentional bias towards human interaction is likely to influence the information the infants have access to, and thus their learning and development. On a shorter timescale, being able to see two interacting agents as more than two detached phenomena is likely to contribute to infant's ability to accurately perceive properties of individual agents' actions as well as properties of the interaction [45]. Having access to this information may help them predict what is going to happen next and proactively adjust their own actions accordingly [46]. Selective attention to others' interactions is also likely to enhance memory of socially important information. Thus, we suggest that the current result describes an adaptive mechanism both on longer and shorter timescales.

Given the role of the STS in both biological motion processing and audiovisual integration [11]; [24]–[25] it is not unlikely that this region contributes to infant's encoding of other people's interactions, as observed in the current study. Moreover, in adults, proactive adjustment while witnessing others' interactions has been linked to brain activity in regions involved in motor and action preparation [47]–.

Reduced preference for biological motion early in life may be a hallmark of autism spectrum disorder (ASD), a neurodevelopmental disorder characterized by deficits in social interaction [49]. Available evidence suggests that already in toddlerhood, children with ASD fail to orient to biological motion representing one individual [50]–[51]. Thus, it can be expected that infants with ASD would not be able to integrate the motion patterns from two agents either, which – assuming functional significance of the current findings – could have cascading developmental consequences. A future direction of the current work could be testing infants at risk for neurodevelopmental problems, particularly in the social domain.

## Supporting Information

Analysis S1 **Supplementary analysis examining Action Type as a factor, examining change across trials with 3 rather than 2 phases, as well as the absolute rather than relative looking measures for the different AOIs.**
(DOCX)

Video S1 **Split-screen video stimulus demonstrating falling action.**
(AVI)

Video S2 **Split-screen video stimulus demonstrating pushing action.**
(AVI)

Video S3 **Split-screen video stimulus demonstrating falling action with the corresponding audio.**
(AVI)

Video S4 **Split-screen video stimulus demonstrating falling action with the corresponding audio.**
(AVI)
