# Supplementary material for: Human Infants Detect Other People's Interactions Based on Complex Patterns of Kinematic Information
Source: PLoS One. 2014 Nov 19;9(11):e112432. doi: 10.1371/journal.pone.0112432 (PMC4237354; doi:10.1371/journal.pone.0112432)
Supplement: Analysis S1 — Supplementary analysis examining Action Type as a factor, examining change across trials with 3 rather than 2 phases, as well as the absolute rather than relative looking measures for the different AOIs. (DOCX) [file pone.0112432.s001.docx]

**Supplementary Analyses**

**Action type**

We investigated the role of Action Type in Study 2 using a 2(Action Type: Falling, Pushing) x 2(Condition: Upright, Inverted) x 2(Phase: Phase I, Phase II) repeated measures ANOVA. There were no main or interaction effects involving Action Type, all *p*s > .05. Moreover, the change from Phase I to Phase II was similar in Falling and Pushing actions. Specifically, in the Upright Condition, there was a significant drop in looking at the non-disrupted interaction when infants were shown the Falling (*M_Phase I_* = .58 *SD _Phase I_* = .22; *M_Phase II_* = .38 *SD_Phase II_*= .25; *t*(25) = 2.56, *p* = .017, *d* = 1.02) and Pushing action (*M_Phase I_* = .61 *SD _Phase I_* = .23; *M_Phase II_* = .39 *SD_Phase II_* = .21; *t*(25) = 2.88, *p* = .008, *d* = 1.152).

In light of this finding we investigated whether the preference for the non-disrupted pair in the Upright Condition was modulated by Action Type in Study 1. Mimicking the negative results in Study 2, no modulation was found in Study 1 either (*F*(1, 27) = 0.759, *ns***)***.*

**Change across trials: splitting into 3 phases**

The current study consisted of only six trials per Condition and Action Type. Due to missing data, splitting into more phases was not possible in the full analysis (doing so would reduce the n to 19). However, to further scrutinize the effect of exposure time in Study 2, we submitted the data from the Upright Condition to a 2(Action Type: Falling, Pushing) x 3 (Phase: Phase I, Phase II, Phase III) repeated measures ANOVA, where Phase I consisted of trial 1 and 2, Phase II of trials 3 and 4 and Phase III of trials 5 and 6. Results revealed a significant effect of Phase (*F*(2, 46) = 5.876, *p*  = .005, *η^2^* = .203), and no other main effects or interaction effects. Inspection of the means indicated a linear trend: preference for the non-disrupted interaction was 0.58 (*SD* = 0.21) in Phase I, 0.48 (*SD* = 0.13) in Phase II and 0.41 (*SD* = 0.25) in Phase III.

**Analysis of absolute measures**

Finally, we investigated the overall looking duration (the sum of looking duration in the screen AOI) in the Upright Condition in Study 2. We found no indication that this measure changed over time, *F*(2, 26) = 1.388, *ns*. Specifically, overall looking duration was 7.22 sec (*SD* = 1.46 sec) for Phase I, 7.44 sec (*SD* = 1.27 sec) for Phase II and 6.97 (*SD* = 1.49 sec) for Phase III. This shows that the change in looking preference was not associated with an overall change in looking time towards the screen. In the Upright Condition, average looking duration to the non-disrupted pair was 4.47 sec (*SD* = 1.97 sec) in Phase I, 3.59 (*SD* = 1.19 sec) in Phase II and 2.31 sec (*SD* = 1.48) in Phase III. Looking time to the disrupted pair was 2.75 sec (*SD* = 1.22 sec) in Phase I, 3.85 sec (*SD* = 1.09 sec) in Phase II and 4.66 sec (*SD* = 2.03 sec) in Phase III.
